# Editorial: Clinically prediction models for gastrointestinal cancer diagnosis and prognosis in the era of precision oncology

**DOI:** 10.3389/fonc.2023.1173367

**Published:** 2023-03-31

**Authors:** Yinyan Gao, Irene XY Wu

**Affiliations:** Xiangya School of Public Health, Central South University, Changsha, China

**Keywords:** prediction model, gastrointestinal cancer, diagnosis, prognosis, precision medicine, oncology

Gastrointestinal cancer remains a major health burden throughout the world, accounting for approximately 26% of all cancer cases and 35% of cancer deaths in 2018 (1). Of these, colorectal cancer and gastric cancer were the second and third leading causes of cancer deaths, respectively (1). The high mortality rates and poor prognosis of gastrointestinal cancers highlight the need for effective strategies to identify high-risk populations and predict prognostic outcomes. Clinical prediction models, which integrate patient data to calculate an individual’s risk (or probability) of either existing diseases (diagnostic models) or future health outcomes (prognostic models), can assist in the early identification of at-risk individuals, facilitating subsequent screening or therapy recommendations (2).

The individual risk estimation of prediction models coincides with the concept of “targeted” in precision oncology, both focus on matching the most accurate and effective strategy for an individual patient. With the aims of providing valuable prediction models for clinical practice and facilitating the advancement of precision oncology, seven studies were collected in the current Research Topic “*Clinical Prediction Models for Gastrointestinal Cancer Diagnosis and Prognosis in the Era of Precision Oncology*”. Specifically, these studies addressed the following issues: (1) constructing and validating a prediction model for a more specific subtype of gastrointestinal cancer (such as gastroenteropancreatic neuroendocrine carcinomas (GEP-NECs), stage II–III gastroesophageal junction adenocarcinoma, among others) (Chen et al., Zuo et al., Hu et al., Huang et al. and Hou et al.); (2) assessing the diagnostic or prognostic values of genetic variables for gastrointestinal cancer (Huang et al. and Zhao et al.); (3) evaluating the prognosis of gastrointestinal cancer when receiving a particular therapy (i.e. neoadjuvant or adjuvant radiotherapy, radiation therapy) (Zuo et al. and Li et al.).

Gastrointestinal cancers encompass a wide range of tumor subtypes. In the past two decades, most prediction models focused on all types of colon cancer or gastric cancer, while few differentiated the specific subtypes. It is generally believed that there are variations in terms of clinical features, prognostic factors and prognostic outcomes among different subtypes of gastrointestinal cancer. A unified prediction model may lead to the misclassification of diagnostic or prognostic outcomes, which may in turn cause inappropriate treatment decisions. Subtype-specific prediction models may provide a more accurate diagnosis and prognosis prediction. Among these seven studies, five prediction models were developed for a specific subtype of gastrointestinal cancer.

Neuroendocrine carcinoma (NEC) is a rare while highly malignant tumor, with the gastroenteropancreatic (GEP) system being one of the most common primary sites. Chen et al. developed a prognostic model for GEP-NEC by incorporating eight demographic and clinicopathological predictors. Excellent discriminative performance has been achieved, with the area under the receiver operating characteristic curve (AUC) ≥ 0.8. Considering the effects of laterality on survival, Hu et al. developed stage-specific prognostic models by incorporating lateral and other clinical information for stage I/II and stage III colon cancer respectively. These two models showed better discrimination than the unified model. Similarly, Zuo et al., Huang et al. and Hou et al. developed prediction models for subtype-specific or clinical feature-specific gastrointestinal cancers.

With the emphasis on genetics in the era of precision oncology, an increasing number of prediction models were developed by including genetic factors to improve predictive accuracy. Huang et al. demonstrated that DNA methylation patterns combined with mutation burden could serve as a novel diagnostic and prognostic biomarker for colorectal cancer. Zhao et al. developed a prognostic model for gastrointestinal stromal tumors by including genetic variables (i.e. fraction genome altered (FGA) score and copy number alteration burden) as candidate predictors, with FGA and tumor mutation burden being included in the final model that showed good predictive performance.

Different therapies for gastrointestinal cancer may vary in prognosis. Zuo et al. identified patient profile as a key explanation for the observed difference in the effects of neoadjuvant or adjuvant radiotherapy and suggested clinicians consider patients’ profiles when selecting therapies. Li et al. explored the relationship between radiotherapy for primary pelvic cancer and subsequent secondary bladder cancer, and observed that radiotherapy is a significant predictor for secondary bladder cancer.

Although these seven articles provided valuable prediction tools for varied gastrointestinal cancers, important limitations remain. According to the Prediction model study Risk Of Bias Assessment Tool (PROBAST), almost all seven prediction models presented methodological limitations in the analysis domain (such as insufficient sample size, inappropriate methods in handling the missing data, or predictor selection). Meanwhile, about half of them did not conduct any external validation, provide calibration results, report final models, and so on. The above issues were also mentioned in previous systematic reviews of prediction models for gastrointestinal cancer (3–5). In addition, only one article followed the TRIPOD (Transparent Reporting of a multivariable prediction model for Individual Prognosis Or Diagnosis), a reporting guideline for prediction models.

We wish to further emphasize that caution should be taken when considering genetic information in prediction models. Adding genetic variables on top of traditional variables in a prediction model does not necessarily improve model performance. A recent meta-analysis of 14 prediction models showed that compared with non-single nucleotide polymorphisms (SNP) models, models that included SNPs had a pooled estimate of 0.040 (95% confidence interval: 0.035-0.045)) AUC improvement (3). Furthermore, no significant correlation was found between the number of SNPs added to the model and the discrimination improvement. In addition, researchers need to take into account that prediction models incorporating genes are undoubtedly costlier, which will hamper their widespread application in practice.

Developing a prediction model is both a science and an art. Despite limitations, we hope the current Research Topic has shed some light on both clinical practice and research in the era of prediction models for gastrointestinal cancer by presenting the articles collected.

## Author contributions

YG summairzed and appraised the collected articles, and wrote the editorial with the input from IW. All authors contributed to the article and approved the submitted version.
